# Efferocytosis in the Central Nervous System

**DOI:** 10.3389/fcell.2021.773344

**Published:** 2021-12-03

**Authors:** Jiayi Zhao, Weiqi Zhang, Tingting Wu, Hongyi Wang, Jialiang Mao, Jian Liu, Ziheng Zhou, Xianfeng Lin, Huige Yan, Qingqing Wang

**Affiliations:** ^1^ Department of Anesthesia, Zhejiang Hospital, Hangzhou, China; ^2^ The First Affiliated Hospital, Wenzhou Medical University, Wenzhou, China; ^3^ Department of Orthopaedic Surgery, Sir Run Run Shaw Hospital, Zhejiang University School of Medicine, Hangzhou, China

**Keywords:** efferocytosis, apoptosis, homeostasis, inflammation, central neural system

## Abstract

The effective clearance of apoptotic cells is essential for maintaining central nervous system (CNS) homeostasis and restoring homeostasis after injury. In most cases of physiological apoptotic cell death, efferocytosis prevents inflammation and other pathological conditions. When apoptotic cells are not effectively cleared, destruction of the integrity of the apoptotic cell membrane integrity, leakage of intracellular contents, and secondary necrosis may occur. Efferocytosis is the mechanism by which efferocytes quickly remove apoptotic cells from tissues before they undergo secondary necrosis. Cells with efferocytosis functions, mainly microglia, help to eliminate apoptotic cells from the CNS. Here, we discuss the impacts of efferocytosis on homeostasis, the mechanism of efferocytosis, the associations of efferocytosis failure and CNS diseases, and the current clinical applications of efferocytosis. We also identify efferocytosis as a novel potential target for exploring the causes and treatments of CNS diseases.

## Introduction

The central nervous system (CNS) accepts afferent information from the whole body, integrates and processes this information into coordinated motor transmission, and stores the information as the basis of memory. Due to the aging population, increases in life stressors and accidents (car accidents, etc.), the incidence rates of CNS diseases, including Alzheimer’s disease (AD), Parkinson’s disease (PD), multiple sclerosis, stroke, epilepsy, and tumors, has increased in recent years (Marino et al., 2007). While substantial attention has been given to studying the CNS, satisfactory treatments for many diseases are lacking due to the complexity of the CNS.

**FIGURE 1:**
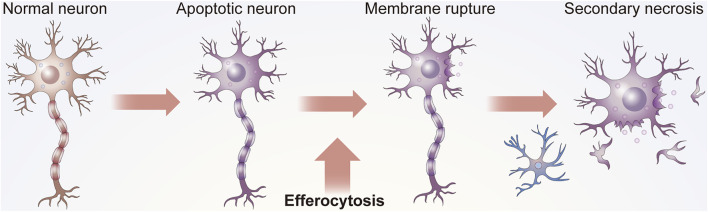
Function of efferocytosis. As shown in Panel 1, efficient efferocytosis can cause ACs, usually apoptotic neurons or glial cells, in the nervous system to be removed by phagocytes to prevent secondary necrosis, the release of cytotoxic substances and damage to surrounding tissues. In addition, some efferocytosis cells present antigens. Efferocytes can distinguish the state of engulfed cells, such as whether they are infected by bacteria or have malignant changes, to subsequently send appropriate signals to the immune system.

Apoptosis is involved in the development and maintenance of homeostasis. This process occurs throughout life in the majority of tissues and is especially crucial in the CNS (Yan et al., 2021; Levine and Kroemer, 2008). As cells are constantly replaced (Taylor et al., 2008), aging and damaged cells need to be removed from the surrounding environment in time to avoid inflammation or tissue damage (Kumar and Birge, 2016) (Figure 1). The elimination of ACs, a process referred to as efferocytosis, requires the phagocytosis of apoptotic cells (ACs) by efferocytes to thereby inhibit excessive inflammation (Zhang et al., 2019a).

Efferocytosis is necessary for maintaining tissue homeostasis under normal physiological conditions and for restoring homeostasis after the occurrence of disease (Doran et al., 2020). ACs are specifically recognized by efferocytes and subsequently engulfed (Hanayama et al., 2004). Without proper efferocytosis, ACs can undergo secondary necrosis, which may lead to the release of dangerous autoantigens in the tissues (Kumar and Birge, 2016). Microglia are considered vital professional efferocytes of the CNS that can clear dying neurons from the CNS (Cai et al., 2019). In some cases, astrocytes and other cells may also have efferocytotic functions.

At present, the role of efferocytosis in the CNS represents a new research direction, as efferocytosis failure has been suggested to be highly related to some diseases, such as stroke, PD, and tumors. Here, we discuss the general mechanisms of efferocytosis, the roles of these mechanisms in the CNS, and their relevance to CNS diseases.

## Efferocytosis in the Central Nervous System

Naturally occurring cell renewal is essential for normal development, tissue homeostasis, and defense against pathogens, and this process is inseparable from a functional efferocytosis mechanism (Morioka et al., 2019). The CNS is equipped with a diffuse and effective network of efferocytes (Maruoka and Suzuki, 2021).

In the CNS, microglia play a major role in the process of removing excess new cells produced during development and cells that die during aging and the development of neurodegenerative diseases (Marín-Teva et al., 2004; Colonna and Butovsky, 2017). Other types of cells, such as astrocytes, neural stem cells, and neural crest cells, can also function as efferocytes (Doran et al., 2020) but are usually less efficient; thus, microglia are considered to be the “professional” efferocytes of the CNS (Borggrewe et al., 2020). During trauma (breach of the blood-brain/blood-spinal cord barrier), the CNS is infiltrated by blood-derived monocytes (Floris et al., 2004). At present, observing ACs in the CNS is difficult under normal circumstances, as ACs are quickly engulfed due to the high efficiency of microglial efferocytosis (Sierra et al., 2010).

However, efferocytosis may be impaired and contribute to the pathologies of CNS diseases due to a combination of aging, inflammation, and specific genetic risk variants. Several studies have indicated that the dysregulation of microglial/macrophage activity is a major cause of some pathological conditions (Márquez-Ropero et al., 2020; Hall-Roberts et al., 2021), such as AD, stroke, amyotrophic lateral sclerosis (ALS), and PD. The mainstream view is that defective efferocytosis results in the inefficient clearance of ACs. The subsequent excessive AC accumulation exacerbates the overloading of the efferocytosis capacity of microglia/macrophages, constituting a vicious feedback loop. The secondary necrosis of ACs that are not cleared in a timely manner leads to the release of proinflammatory factors, intracellular molecules and danger-related molecular patterns (DAMPs), thereby intensifying the damage (Flannagan et al., 2012; Mike and Ferriero, 2021).

In addition, previous studies of the nervous system have more broadly summarized phagocytosis (Brown and Neher, 2014). Here, we distinguish between the concepts of phagocytosis and efferocytosis. The term phagocytosis (derived from phagos, which means eating, and cyte, which means cell) describes the process by which a cell recognizes, engulfs and digests a target that is ≥ 1 μm in size (for example, living cells, dead cells, dying cells, and larger fragments), while efferocytosis refers to the removal of only dead/dying cells. The term “efferocytosis” is now used to distinguish the phagocytosis of ACs from other phagocytic processes (Fu et al., 2014).

In short, the efficiency of efferocytosis determines the dynamics of apoptosis during development and disease.

## Recognition of Efferocytosis

Efferocytosis is a multistep process involving recognition, engulfment and digestion; thus, a set of signaling molecules likely plays important roles at each stage. The process of efferocytosis is typically universal across the nervous system, but some distinctions are evident. Therefore, we next cover the basics of the common signals in efferocytosis and their association with the nervous system.

### “Find me” Signals

**FIGURE 2:**
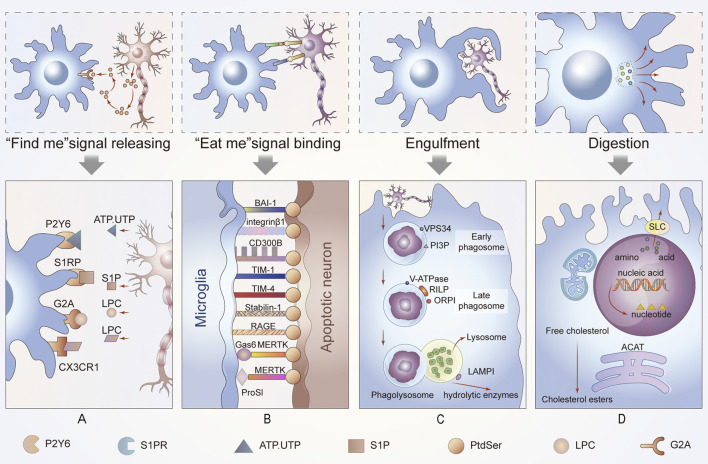
Process of efferocytosis and related changes. In this part of the diagram, we show the general process of efferocytosis in the nervous system. A series of molecules attract phagocytes to ACs and bind to them, and the membranes of the microglia, the resident phagocytes of the CNS, then stretch out and wrap around apoptotic neurons or glia until they are engulfed. The dead cells are gradually digested and excreted in the maturing phagosomes. This process is accompanied by energy metabolism and anti-inflammatory metabolism. **(A)** Newly ACs, still morphically normal, emit a “find me” signal that binds to different receptors on nearby microglia, enticing these cells to migrate to ACs to perform their function. **(B)** When microglia come into contact with ACs, they interact with cell receptors that function as “eat me” or “do not eat me” signals to determine their subsequent function. **(C)** Phagosomes gradually mature until they bind to lysosomes. This process is carried out under the premise of vps34, phosphotidylinositol-3-phosphate (PI3P) and Rab protein activation. Additionally, the acidity of the phagosome increases. **(D)** Finally, the contents of phagocytes are degraded or discharged after treatment in different ways. If cholesterol is esterified into cholesterol esters, amino acids are discharged through the solute carrier family. These processes are also accompanied by changes in intracellular energy or other aspects of metabolism, such as mitochondrial fission and fusion.

“Find me” signals regulate the activity of macrophages alone or crosstalk among other molecules. Microglia migrate to stroke foci in a timely manner and initiate phagocytosis on the first day after a stroke, which improves the prognosis of the afflicted patient. Further studies revealed that the recruitment of phagocytes to ACs is triggered by the “find me” signal of ACs (Schilling et al., 2005). Injured or dying cells release “find me” signals at the earliest stages of apoptosis either directly or indirectly, and the signals form a gradient within the tissue. The “find me” signals are sensed by receptors on by adjacent efferocytes, causing them to migrate to dying cells. This process has been fully confirmed in many neurological diseases, including demyelinating diseases, such as multiple sclerosis. The timepoint at which the mediators are released ensures that cells can be engulfed before secondary necrosis to avoid neuroinflammation (Ravichandran, 2011). The recognition of these signals is sensitive, and this process is necessary for the efficient clearance of cells from the nervous system (Peter et al., 2010) (Figure 2A).

A set of signaling molecules, including lyso-phosphatidyl choline (LPC), fractalkine (CX3CL1), sphingosine-1-phosphate (S1P), and nucleotides ATP and UTP, has been proposed to regulate efferocyte migration (Medina and Ravichandran, 2016). These molecules function by binding to their respective receptors. The characteristics of these signals and their receptors are listed in the table below (Table 1). Next, we explore the special role of these “find me” signals in the nervous system (Gude et al., 2008; Truman et al., 2008).

**TABLE 1:** Some signaling molecules and receptors in the process of efferocytosis. “find me” signals “eat me” signals.

**Find me**							
**Ligands**	**Receptors**	**Macrophages function**	**Refs**	**Microglia function**	**Refs**	**Associated diseases**	**Refs**
UTP, ATP	P2Y6	Inducing the directional migration of phagocytes	Chiu et al. (2017)	Inducing the directional migration of phagocytes	Dahl and Muller (2014)	Multiple sclerosis, experimental allergic encephalomyelitis	Lutz et al. (2013)
S1P	S1PR		Gude et al. (2008), Liao et al. (2018)		Gude et al. (2008)	Systemic Lupus Erythematosus	Mike et al. (2018)
LPC	G2A		Medina and Ravichandran (2016)		Medina and Ravichandran (2016)	Autoimmunity, colitis	Frasch et al. (2016), Le LQ et al. (2001)
CX3CL1	CX3CR1	Regulating the activity of macrophages	Truman et al. (2008), Tsai et al. (2021), Ziegenfuss et al. (2008)	Regulating the activity of macrophages	Chamera et al. (2020)	Atherosclerosis	Liu and Jiang (2011)
**Eat me**							
**Ligands**	**Receptors**	**Macrophages function**	**Refs**	**Microglia function**	**Refs**	**Associated diseases**	**Refs**
PS	BAI-1	Phagocytosis of apoptotic cells	Murakami et al. (2014a)	Phagocytosis of apoptotic cells	Murakami et al. (2014a)	Learning and memory	Duman et al. (2016)
	Integrinβ1		Schittenhelm et al. (2017)		Schittenhelm et al. (2017)	Autoimmunity	Schittenhelm et al. (2017)
	CD300b		Murakami et al. (2014b)	Not expressed in microglia	Gasiorowski et al. (2013)	Inflammatory Bowel Disease	Tian et al. (2014)
	TIM-1		Freeman et al. (2010)	Phagocytosis of apoptotic cells	Freeman et al. (2010)	Kidney ischemia/reperfusion injury	Yamanishi et al. (2010)
	TIM-4					Ischaemia–reperfusion injury, Systemic Lupus Erythematosus	Li and Weinman, (2018)
	Stabilin-1		Kim et al. (2012)	Function in phagocytosis unreported		Glomerular fibrosis	Schledzewski et al. (2011)
	Stabilin-2						
	RAGE		Schledzewski et al. (2011)	Phagocytosis of apoptotic cells	Xie et al. (2013)	Sepsis, ibrosis, allergic airway inflammation, atherosclerosis, Muscle regeneration	Nienhuis et al. (2009), Riuzzi et al. (2018), van Zoelen and van der Poll (2008)
ProS1	Mertk		Geng et al. (2017)		Geng et al. (2017)	Systemic Lupus Erythematosus, Autoimmunity, Colon cancer, Arthritis, Parkinson disease, multiple sclerosis, experimental allergic encephalomyelitis	Miyanishi et al. (2012), Waterborg et al. (2018), Wium et al. (2018)
Gas6			Happonen et al. (2016)		Grommes et al. (2008)	Ischaemia–reperfusion injury, Melanoma	Bellan et al., (2019), Wu et al. (2017)

S1P selectors and antagonists have been suggested to alleviate neuroinflammation and multiple sclerosis and can likely serve as therapeutic targets in autoimmune diseases. Based on with the close relationship between efferocytosis and autoimmune diseases, we speculate that S1P plays an important role in nervous system diseases as a “find me” signal (Tsai and Han, 2016).

Nucleotides can also serve as “find me” signals. ATP and UTP are released as “chemotactic” extracellular alarmins rather than by “chemokinesis” and are sensed by purinergic P2Y receptors, inducing the directional migration of efferocytes (Chiu et al., 2017). This secretion of ATP and UTP is governed by pannexin 1 (PANX1), a plasma transmembrane protein, and defects in this protein can result in multiple sclerosis and experimental allergic encephalomyelitis (Lutz et al., 2013).

“Find me” signals have additional roles. In some organs and tissues (such as the thymus), ACs are engulfed by resident efferocytes, and there is thus no need to recruit proximate efferocytes. In this situation, the “find me” signal is still released to regulate the activity of macrophages (Ziegenfuss et al., 2008). This study was not conducted on the nervous system, but we speculate that the “find me” signal also helps to regulate the activities of efferocytes and microglia in the nervous system.

### “Eat me” Signals

When efferocytes are in sufficiently close proximity to perform efferocytosis, “eat me” signals are used to distinguish live cells from dead cells. “Eat me” signals are a set of cell-surface molecules that facilitate the next step of cell engulfment (Figure 2B).

These “eat me” signals include exposure to PtdSer, changes in charge and glycosylation patterns on the cell surface, alterations in intercellular adhesion molecule-1 (ICAM-1) epitopes on the cell surface, and exposure to the endoplasmic reticulum-resident protein calreticulin (Nagata et al., 2010). One of the most widely studied “eat me” signals is exposure to PtdSer.

PtdSer is commonly expressed on the inner leaflet of the plasma membrane of healthy cells and seldomly in the outer leaflet. PtdSer concentrations change on the outer leaflet and provide “specificity” for efferocytes; thus, they are advantageous for the recognition of ACs (Segawa et al., 2018). The recognition of phosphatidylserine by efferocytes is also regulated by the threshold of phosphatidylserine exposure (Borisenko et al., 2004). In a hypoxic environment caused by ischemic stroke, microglia may phagocytize cells that are not entirely apoptotic by recognizing everted PtdSer. In addition, everted PtdSer may help efferocytes phagocytize living cells (Brelstaff et al., 2018).

Studies have revealed other receptor systems that participate in the PtdSer direct binding and recognition of molecules such as integrinβ1, the single immunoglobulin-domain type I transmembrane protein CD300b, and brain-specific angiogenesis inhibitor 1 (BAI1). CD300b is expressed by neutrophils and peritoneal macrophages but not microglia. It can recognize phosphatidyl-serine (PS) and phosphatidyl-ethanolamine (PE), the knockdown of which results in efferocytosis impairment (Murakami et al., 2014). In addition, BAI1 may emit different types of signals in efferocytes, which potentially contributes to signal amplification and allows efferocytes to identify apoptotic neurons or other cells (Park et al., 2007).

The “eat me” signal can also induces the phagocytosis of delimited cell surface domains by microglia. This local “eat me” signal enables efferocytes to precisely determine how much of the outer membrane needs to be “engulfed”. Synaptic pruning defects associated with complement deposition may lead to neurodegenerative diseases and neuropsychiatric diseases (Barclay and Van den Berg, 2014).

Whether other possible “eat me” signals promote efferocytosis has not been studied in detail in the nervous system.

### The “do not Eat me” Signal

In contrast to ACs, normal tissues express a “do not eat me” signal, namely, an anti-efferocytosis signal that helps healthy cells escape macrophage-mediated efferocytosis. These signals are hidden in ACs. Some of these signals, such as CD24, may also help tumor cells escape efferocytosis, leading to peripheral tissue damage (Gardai et al., 2006). CD24 is a marker of human glioma that can help tumor cells escape phagocytosis and worsen the disease (Poncet et al., 1996). CD24 also plays such a role in the nervous system.

Recently, the “do not eat me” CD24 signal was shown to interact with the inhibitory receptor sialic acid-binding Ig-like lectin 10 (Siglec-10) on tumor-associated macrophages (TAMs), orchestrating a novel innate immune checkpoint. Additionally, CD47 has been found to bind to signal regulatory protein-α (SIRPα) on macrophages to inhibit phagocytosis (Bradley, 2019). The expression of CD47 prevents neuronal injury resulting from the extravagant pruning of synapses by microglia in the nervous system (Lehrman et al., 2018). However, studies have revealed that the role of CD47 as a “do not eat me” signal is limited (Barclay and Van den Berg, 2014).

## Engulfment and Digestion in Efferocytosis

The process of efferocytosis and digestion can be divided into the following three parts: efferocytes phagocytize apoptotic neurons or other cells to form phagosomes, the phagosomes mature and combine with lysosomes, and finally, the phagosome is degraded, thereby completing efferocytosis. In the nervous system, any obstruction of these steps may cause nervous system diseases (Figures 2C,D).

### Coordinated Engulfment of Dead and Dying Cells

After recognition and binding to ACs, the efferocytes rearrange the membrane by actin aggregation. The membrane is partially trapped and expands to surround ACs and efferocytes, forming a phagosome (Richards and Endres, 2014).

The synergism of actin depolymerization and dynein plays an important role in the shedding and division of phagosomes (Liao et al., 1992; Nienhuis et al., 2009).

An indispensable step in the formation of phagosomes is the sealing of the phagosomal membrane. The blockade of phagosomes may be related to the activation of myosin by cytosolic calcium, which is PLCc dependent. Phagosome closure can also be promoted by BAR domain-containing proteins through actin-directed polymerization and membrane deformation (Linkner et al., 2014). Experiments have indicated that microglia, which play a role in efferocytosis in the nervous system, need to form a closed space to successfully complete the efferocytosis process (Samejima and Earnshaw, 2005).

### Maturation of the Phagosome

The maturation process after phagosome formation is distinguished and defined by different biochemical markers of initial phagosomes and late phagosomes (Flannagan et al., 2012). For example, Rab5 is a marker of early phagosomes, whereas acidity is significantly enhanced in late phagosomes. Phagosomes mature due to the actions of a series of molecules, including VPS34, phosphatidylinositol-3-phosphate (PI3P) and RAB proteins (Vieira et al., 2001). However, some differences exist in the formation of phagosomes in different efferocytes in terms of pH, surface proteins and other factors (Henry et al., 2004). Reactive oxygen species (ROS) are released due to the actions of the NADPH oxidase family in efferocytes, representing the anti-inflammatory mechanism of efferocytes, which is significant for avoiding neural inflammation (Nunes et al., 2013).

### Breakdown of Phagolysosomal Contents

The combination of phagosomes and lysosomes begins with the formation of phagosomes (Rubino et al., 2000). Lysosomes contain a variety of enzymes, such as lysosomal lipases, cathepsins, cationic peptides and hydrolytic enzymes, which can hydrolyze efferocytes. Phagosomes can be modified to regulate their fusion (Levin et al., 2016).

In contrast to the formation of phagosomes and their fusion with lysosomes, the details of phagosome degradation have not been studied clearly, especially in the nervous system. Recently, the formation of a closed bag structure, the gastrosome, during efferocytosis by microglia was shown to be essential for efficient efferocytosis and digestion. The gastrosome is likely involved in membrane circulation or metabolism during efferocytosis (Villani et al., 2019).

To prevent macromolecules from apoptotic neurons or other cells from adversely affecting efferocytes and the neural microenvironment, efferocytes must metabolize these residues from apoptotic neurons or other cells. The contents of efferocytes are hydrolyzed by these enzymes and released after decomposition to avoid neuroinflammatory reactions.

Nucleic acids are partially degraded by nucleases in dying cells, and this process continues in efferocytes. Deoxyribonuclease II (DNase II) is the main nucleic acid-degrading enzyme in efferocytes. This degradation process is critical for dealing with the nuclei that are extracted by red blood cells (Mellors et al., 1975; Samejima and Earnshaw, 2005).

In addition, esterified cholesterol is hydrolyzed by hydrolase, while unesterified cholesterol is discharged from the compartment to prevent accumulation (Friedland et al., 2003; Trost et al., 2009). Lysosomal phospholipase A2 (LPLA2) cleaves glycerophospholipids, which are excreted from phagosomes by unknown mechanisms (Fischer et al., 2001). Cholesterol disorders have been shown to be closely related to peripheral nerve injury, Huntington’s disease and PD.

Proteins present at high concentrations in each physiological phagocytic target region are degraded into polypeptide chains or amino acids by different protease subfamilies (such as serine, aspartic acid and cysteine proteases) at different stages of efferocyte maturation (Kinchen and Ravichandran, 2008). The solute carrier family plays an important role in the transmembrane transport of amino acids, the role of which in the nervous system cannot be underestimated (Abraham, 2015). Incidentally, intracellular efferocytosis clears beta-amyloid, and failure to do this is a known cause of AD symptoms (Tajbakhsh et al., 2021).

In addition, amino acid metabolism is indispensable in nervous system efferocytosis. Efferocytosis enables macrophages to take up arginine and ornithine from apoptotic neurons or other cells and convert them into putrescine. Putrescine activates Rac1 and promotes subsequent rounds of efferocytosis. Failure of this pathway is likely related to neurodegenerative diseases (Yurdagul et al., 2020). Other studies have shown that nervous system damage is a consequence of arginine deficiency, such as neurotoxicity, through oxidative damage or neuronal demyelination induction. This may be explained by efferocytosis (Wiesinger, 2001).

Lack of the cationic amino acid transporter SLC7A7 hinders efferocytosis. SLC7A7 loss results in the serious accumulation of cationic amino acids in efferocytosis. In humans, SLC7A7 gene deficiency can lead to lysine protein intolerance (LPI). In addition to alveolar protein accumulation and hemophagocytic lymphohistiocytosis, over half of LPI patients have cognitive impairment (Wiesinger, 2001), which is probably attributed to microglia being incapable of removing the accumulated nerve cell corpse due to defective gene death occurring simultaneously with developmental neuron death. Additionally, defects in this amino acid transporter have been proven to be related to bubble brain in zebrafish (Torrents et al., 1999). It is worth noting that efferocytosis can be visualized in the brains of living zebrafish; thus, these organisms play an important role studying efferocytosis in the nervous system.

## Effects After Efferocytosis

After the efferocytosis of ACs, a series of corresponding changes must occur in efferocytes to meet the needs of digestion and elimination (Torrents et al., 1999).

Digestion-related endocytosis is an energy-intensive process; additionally, each step of efferocytosis, such as actin rearrangement, requires a sufficient energy supply. Therefore, corresponding changes occur at each metabolic step to provide a sufficient amount of energy for each process (Lin et al., 2021). In addition, efferocytosis has significant anti-inflammatory effects, and a series of anti-inflammatory changes during metabolism greatly contribute to the immune-silencing effects of efferocytosis (Tabas and Bornfeldt, 2020).

### Energy Metabolism

Other changes are also required to meet the high energy demands required for efferocytosis. The dependence of efferocytes on glycolysis is increased after efferocytosis, which is determined by the demand for energy and the anti-inflammatory effect of the glycolysis product lactate on surrounding cells (Morioka et al., 2018).

The first change is the activation of NADPH oxidase. Efferocytosis can effectively activate NADPH oxidase in a cd11b-, tlr2-, TLR4-or MyD88-dependent manner. NADPH oxidase plays a key role in the clearance of apoptotic neurons by inflammatory macrophages. The generated oxidants promote efferent maturation and acidification. Intraluminal acidification is essential for the effective digestion of different bodily proteins, and the degradation level of ingested apoptotic neurons or other cells is improved by this process (Bagaitkar et al., 2018; Schulz et al., 2021).

In addition, when exposed to apoptotic neurons, the adenylate-activated kinase AMPK is activated due to the inhibition of mitochondrial oxygen consumption and ATP output (Jiang et al., 2013).

Both of these steps produce ROS, which can enhance efferocytosis. ROS formation is also regulated by inositol phosphate 3-phosphate bound to the p40phox oxidase subunit. ROS formation has been shown to be associated with efferocytosis in autoimmune diseases but is known only to promote neuronal apoptosis in the nervous system (Lin et al., 2021).

Sustained efferocytosis requires mitochondrial division mediated by dynamic associated protein 1 (drp1), which is triggered by the uptake of apoptotic neurons or other cells. Hindering mitochondrial division passivates the increase in cytoplasmic calcium, which leads to the weakening of calcium-dependent phagosome formation during the subsequent efferocytosis of apoptotic neurons or other cells; thus, the phagocytic ability will naturally decline, including in the nervous system (Wang et al., 2017). Studies have also shown that mitochondrial division and fusion are pathogenic features of AD (Horst et al., 2019).

### Anti-Inflammatory Metabolism

The immune-silencing effect of efferocytosis is mainly achieved by the upregulation of anti-inflammatory factors and the downregulation of pro-inflammatory factors (Tabas and Bornfeldt, 2020).

As mentioned above, the level of glycolysis is increased, and its byproduct lactate provides an anti-inflammatory environment for surrounding cells (Morioka et al., 2018).

In a metabolome enrichment analysis, the concentrations of long-chain free fatty acids and fatty acids were shown to be increased significantly, which stimulated an increase in the expression of the anti-inflammatory factor interleukin-4 (Horst et al., 2019).

Research has also proven that lipid metabolism causes the mitochondrial electron transport chain to release NAD+ and leads to an increase in the expression of interleukin-10, which is a significant anti-inflammatory signal (Horst et al., 2019). The relationship between oxidative stress and neuroinflammation has been reported in the literature (Bhowmick et al., 2019).

Fatty acid-derived specialized proinflammatory mediators (SPMs) are also special anti-inflammatory mediators that have certain neuroprotective effects. When ACs are ingested by macrophages, SPM precursors in AC microbubbles are transformed into mature lipid mediators, and proinflammatory leukotriene synthesis is reduced (Doran et al., 2020).

Defective anti-inflammatory metabolism may lead to CNS diseases, including stroke, AD and PD (Hall-Roberts et al., 2021). This will be discussed in detail in the next part of this paper. Additionally, the proinflammatory effect of efferocytosis defects may also be caused by the secondary necrosis of apoptotic neurons or other cells that have not been cleared in a timely manner (Kawano and Nagata, 2018).

In addition to anti-inflammatory effects, efferocytosis typically also generates pro-inflammatory products, such as excessive ROS, as mentioned above.

In short, studies have shown that a series of immune processes typically protect cells from neuroinflammation during efferocytosis. This is a good explanation for why, in some diseases, such as the neuroinflammatory disease AD, efferocytosis abnormalities are likely to become new treatment targets.

## Nervous System Diseases Related to Efferocytosis

As mentioned above, renewal of the cell cycle not only affects normal nervous system development and homeostasis maintenance but has also been observed in neurodegenerative diseases and neurological diseases, such as PD, epilepsy, stroke, and tumors.

### Ischemic Stroke

To date, little is known about the cellular and molecular mechanism of efferocytosis after ischemic stroke and its impact on injury and recovery. The phagocytic activity of efferocytes may differ at different stages of injury after ischemic stroke (Ting et al., 2020) and may change due to biological variables such as age and sex. These are critical factors to consider when developing efferocytosis‐manipulating strategies.

Damaged neurons after stroke release DAMPs to initiate a series of inflammatory responses and thereby limit damage (Liu et al., 2021). Efferocytes quickly process dead cells and induce a relaxed microenvironment that promotes repair and regeneration. However, the clearance of apoptotic neurons in a mouse model of stroke-induced cerebral artery occlusion (MCAO) is extremely poor (Faustino et al., 2011). Persistent dead/dying neurons can cause excessive inflammation, exacerbate secondary damage, and hinder repair.

However, a controversial role for efferocytosis in the poststroke brain has also been proposed, wherein efferocytes attack viable neurons or oligodendrocyte precursor cells and exacerbate brain injury after cerebral ischemia (Neher et al., 2013).

### Cerebral Hemorrhage

Studies have shown that monocyte-derived macrophages (MDMs) are essential for optimal hematoma removal and nerve recovery (Chang et al., 2018).

Physiologically, macrophages efficiently remove apoptotic erythrocytes from the peripheral circulation to prevent inflammation. A similar process has been observed in the brain after hemorrhage (Chang et al., 2018). The efferocytosis of erythrocytes by MDMs can lead to the removal of hematomas and reduce secondary damage.

The efferocytosis of macrophages can regulate the macrophage phenotype and promote recovery through AXL/MERTK (Grabiec et al., 2018; Myers et al., 2019). Gas6, the adaptor protein of AXL, has been shown to reduce brain edema and improve behavioral abilities in mice after intracerebral hemorrhage (Tong et al., 2017).

### Glioblastoma

GBM is a common malignant primary brain tumor with a poor prognosis (Wu et al., 2021a).

Regulating the immunosuppressive microenvironment of GBM, such as by targeting glioma-associated macrophages and microglia, could be a potential alternative treatment method (Desbaillets and Hottinger, 2021). Studies have shown that efferocytosis is closely related to the polarization of the M1/M2 phenotype. Unlike nonneoplastic diseases, the inflammatory defense function of macrophages/microglia can be inhibited in the glioma microenvironment, where they cells are changed from the M1 phenotype (proinflammatory) to the M2 phenotype (anti-inflammatory) (Calero-Pérez et al., 2021). The mainstream view is that anti-inflammatory substances released after efferocytosis induce microglia to polarize to the M2 type. These findings suggest that regulating efferocytosis function may be an alternative treatment strategy (Gjorgjevski et al., 2019). In addition, studies have demonstrated that GBM is closely related to the overexpression of the “do not eat me” signal CD47, which allows the cells to escape efferocytosis (Hu et al., 2020; Wu et al., 2021b).

### Parkinson’s Disease

PD is another example in which the phagocytic behavior of microglia is altered. The most important pathological change in PD is the significant decrease in the dopamine (DA) content in the striatum, which is caused by the death of dopaminergic neurons in the substantia nigra of the midbrain (Fang et al., 2015). This result suggests that the excessive efferocytosis of dopaminergic neurons in the substantia nigra is an important mechanism in the pathological process of PD (Fourgeaud et al., 2016; Tremblay et al., 2019).

As efferocytosis is a new concept proposed only recently and the nervous system is extremely complex, sufficient data are lacking; therefore, in-depth conclusions on the role of efferocytosis in neurological diseases cannot be drawn. However, we believe that studying efferocytosis will provide new insight into the mechanisms and treatments of neurological diseases.

In addition to the diseases described above, in pathologies such as epilepsy, the increase in the number of early ACs is not due to apoptosis induction but rather to the accumulation of impaired phagocytic function and unremoved ACs (Abiega et al., 2016). In addition, efferocytosis dysfunction has been observed in traumatic CNS diseases (Milich et al., 2019).

## Methods of Studying Efferocytosis

To facilitate more in-depth research on efferocytosis, we compiled some of the methods and tools currently used in efferocytosis research.

It is common to coculture efferocytes and ACs and then assess the efficiency of efferocytosis with a fluorescence microscope or flow cytometer (Proto et al., 2018; de Couto et al., 2019) to, for example, analyze the number of uncleared ACs after coculture (Cai et al., 2019). It should be pointed out that studies often uses specially treated silica beads to simulate ACs (Yin et al., 2016), while other studies detect the efficiency by BrdU labeling (Sierra et al., 2010).

Moreover, the expression of various signaling molecules, such as PtdSer, is generally measured by Western blotting or immunofluorescence (Liu et al., 2013), and the corresponding concentrations can also be measured by ELISA (Chang et al., 2018). Immunofluorescence can also be used to observe the process by which ACs are engulfed by efferocytes based on the observance of specific protein markers by fluorescence microscopy (Vieira et al., 2003).

## Conclusion

The timely removal of dead cells is critical for the maintenance of homeostasis and recovery after the occurrence of disease (Zhang et al., 2019b). Additionally, the maintenance and restoration of homeostasis depend on functional efferocytosis in the CNS.

Here, we discuss the occurrence of efferocytosis in the CNS, characteristic proteins as well as energy changes and metabolic changes after efferocytosis. Whether a specialized functional efferocytotic mechanism exists in the nervous system remains unclear. However, researchers have found that efferocytosis by microglial efferocytes is involved in various neurological anomalies, such as stroke, brain trauma, and brain tumors (Márquez-Ropero et al., 2020). However, due to the complexity of the CNS, the dominant efferocytes and main changes in functional efferocytosis under steady-state conditions and various pathological conditions are still unclear. In a stroke study, sequencing revealed no significant differences in the expression of efferocytosis-related genes between microglia and exogenous macrophages (Zhang et al., 2019a).

Combined with the results of studies focusing on other diseases, we found that the type of disease, length of disease, and timepoint at which efferocytosis failure occurs may have different effects on the resulting disease. Additionally, studies on liver diseases have revealed that the excessive inhibition of proinflammatory signals can lead to defects in inflammation regression. The early inflammatory response lays the foundation for the induction of anti-inflammatory and pro-regression pathways (Horst et al., 2019).

Due to its extensive role in the nervous system and significant anti-inflammatory properties, efferocytosis is expected to become a therapeutic target for various neuroinflammatory diseases and other CNS diseases. However, the study of efferocytosis in the nervous system is still in its infancy.

The mechanism of efferocytosis suggests that unblocked signaling pathways, rearrangement of the cell membrane and adequate energy supply are possible causes of efferocytosis disturbance and potential targets of some diseases. In many efferocytosis-related diseases, such as AD and atherosclerosis, substances similar to lipid plaques and α-amyloid accumulate, indicating that the metabolic processes occurring after efferocytosis, especially excretion, play important roles in the disease. The abundance of lipids in the nervous system and the accumulation of myelin after apoptosis urgently need to be resolved. Therefore, in addition to lysosomes responsible for digestion and transport-related membranes, enzymes that play a role in digestion and enrichment are also worthy of attention, as they may provide some implications for studying vacuolar ATPase and other proteins related to lysosomal acidity. The rate of phagocyte recruitment and the duration of their role in efferocytosis may also be worthy of more investigation.

Studies have found that the efferocytosis of apoptotic erythrocytes after intracerebral hemorrhage significantly promotes nerve recovery (Chang et al., 2018). Moreover, efferocytosis can regulate the neuroinflammatory environment in AD and PD, thereby showing excellent therapeutic potential. In addition, the inflammatory microenvironment may change the expression of efferocytosis-related molecules, thus hindering efferocytosis, revealing that anti-inflammatory therapies not targeting efferocytosis may also alleviate inflammation through efferocytosis (Belchamber and Donnelly, 2017). In addition, transformation of the macrophage phenotype to promote efferocytosis also contributes to wound healing (Bäck et al., 2019).

More in-depth studies on the mechanisms of efferocytes in the CNS must be performed to identify the best possible strategies for targeting efferocytes and thereby aid in disease treatment and tissue regeneration.

Azithromycin (Hodge et al., 2008), vitamin A and carotene derivatives (Duquette et al., 2014) have been proven to improve the symptoms of some diseases through efferocytosis in animal models and clinical trials of nonnervous system diseases. However, the applications of potential drugs in the nervous system, in both the blood-brain barrier and the blood-spinal barrier, must be considered. Biomaterials may be used to deliver drugs that target efferocytosis. Sonodynamic therapy (Sun et al., 2019) is a type of tumor therapy, and the synergistic effects of ultrasound and drugs on efferocytosis represents a promising anti-inflammatory mechanism in CNS diseases.

Admittedly, neurological diseases are age-old challenges, but we believe that the research and future application of efferocytosis can be used to remove some of their obstacles.

## GLOSSARY

ATP: Adenosine-triphosphate
BAI-1: Brain-specific angiogenesis inhibitor 1
CD300b: CD300 antigen-like family member B
CX3CL1: Fractalkine
CX3CR1: Fractalkine receptor
G2A G: protein-coupled receptor G2A
Gas6: Growth Arrest Specific Protein 6
Integrinβ1: Integrinβ1
LPC: Lyso-phosphatidyl choline
Mertk: AXl and mer proto-oncogene tyrosine kinase
P2Y6: Purinergic receptor Y6
ProS1: Protein S
PS: Phosphatidylserine
RAGE: Receptor for advanced glycation end products
S1P: Sphingosine-1-phosphate
S1PR: Sphingosine-1-phosphate receptor
Stabilin-1: Common lymphatic endothelial vascular endothelial receptor-1
Stabilin-2: Common lymphatic endothelial vascular endothelial receptor-2
TIM-1: T cell immunoglobulin and mucin domain-containing molecule 1
TIM-4: T cell immunoglobulin and mucin domain-containing molecule 4
UTP: Uridine triphosphate
